# Bladder cancer and occupational exposure to diesel and gasoline engine emissions among Canadian men

**DOI:** 10.1002/cam4.544

**Published:** 2015-10-28

**Authors:** Lidija Latifovic, Paul J. Villeneuve, Marie‐Élise Parent, Kenneth C. Johnson, Linda Kachuri, the Canadian Cancer Registries Epidemiology Group, Shelley A. Harris

**Affiliations:** ^1^ Prevention and Cancer ControlCancer Care OntarioTorontoOntarioCanada; ^2^ CHAIM Research CentreCarleton UniversityOttawaOntarioCanada; ^3^ Occupational Cancer Research CenterTorontoOntarioCanada; ^4^ INRS‐Institut Armand‐FrappierInstitut national de la recherche scientifiqueUniversity of QuebecLavalQuebecCanada; ^5^ School of EpidemiologyPublic Health and Preventive MedicineUniversity of OttawaOttawaOntarioCanada; ^6^ Division of EpidemiologyDalla Lana School of Public HealthUniversity of TorontoTorontoOntarioCanada; ^7^ Division of Occupational and Environmental HealthDalla Lana School of Public HealthUniversity of TorontoTorontoOntarioCanada

**Keywords:** Bladder cancer, Case–control study, engine emissions, expert assessment, job‐exposure matrix, occupational cancer

## Abstract

The International Agency for Research on Cancer has classified diesel exhaust as a carcinogen based on lung cancer evidence; however, few studies have investigated the effect of engine emissions on bladder cancer. The purpose of this study was to investigate the association between occupational exposure to diesel and gasoline emissions and bladder cancer in men using data from the Canadian National Enhanced Cancer Surveillance System; a population‐based case–control study. This analysis included 658 bladder cancer cases and 1360 controls with information on lifetime occupational histories and a large number of possible cancer risk factors. A job‐exposure matrix for engine emissions was supplemented by expert review to assign values for each job across three dimensions of exposure: concentration, frequency, and reliability. Odds ratios (OR) and their corresponding 95% confidence intervals were estimated using logistic regression. Relative to unexposed, men ever exposed to high concentrations of diesel emissions were at an increased risk of bladder cancer (OR = 1.64, 0.87–3.08), but this result was not significant, and those with >10 years of exposure to diesel emissions at high concentrations had a greater than twofold increase in risk (OR = 2.45, 1.04–5.74). Increased risk of bladder cancer was also observed with >30% of work time exposed to gasoline engine emissions (OR = 1.59, 1.04–2.43) relative to the unexposed, but only among men that had never been exposed to diesel emissions. Taken together, our findings support the hypothesis that exposure to high concentrations of diesel engine emissions may increase the risk of bladder cancer.

## Introduction

Bladder cancer is the sixth most common cancer in men worldwide, with an estimated incidence of more than 330,000 new cases a year 1. Most often, bladder cancer starts in the cells of the urothelium; urothelial carcinomas make up ~90% of all bladder cancers in the developed world, with the remainder consisting of squamous cell carcinoma and adenocarcinoma 2. Smoking is the leading risk factor for bladder cancer; however, other environmental and occupational exposures are also known to increase the risk of developing bladder cancer 3. Exposure to aromatic amines (*β*‐naphthylamine, 4‐aminobiphenyl, 4‐chloro‐*o*‐toluidine and benzidine and 4,4′‐methylenebis(2‐chloroaniline)), which can be found in chemical, dye, and rubber industries as well as in hair dyes, paints, fungicides, cigarette smoke, plastics, metals, and motor vehicle exhaust is often cited as the most important occupational risk factor for bladder cancer 2, 3, 4, 5. Otherwise, the most consistent evidence has been found for polycyclic aromatic hydrocarbons (PAHs) 5, 6. An estimated 4–7% of bladder cancers are due to work‐related exposures 6, though historically, this attributable risk was estimated to be 37% among those employed before the 1950s in occupations identified as high risk, such as transport equipment operators, machine assemblers, or mining and quarrymen 6.

Occupational exposure to diesel emissions is widespread affecting an estimated 781,000 Canadian workers, or 5% of the working population, 92% of whom are male 7. In the U.S., an estimated 1.4 million workers are exposed in the workplace 8. Exposure in the general population is also ubiquitous but at lower levels. Engine emissions are composed of a complex mixture of gases and fine particles, the composition of which includes elemental carbon with a surface coating of sulfates, nitrates, and other trace elements as well as known carcinogens such as PAHs, nitroarenes, phenols, and heterocyclic compounds 9. Many other known or suspected carcinogens and mutagens are adsorbed to the exhaust particle's surface 9. Diesel‐ and gasoline‐powered engines emit gases such as oxides of carbon, nitrogen, and sulfur as well as low molecular weight hydrocarbons.

In 2012, the International Agency for Research on Cancer (IARC) classified diesel emissions as carcinogenic to humans (Group 1), based on evidence for lung cancer, an update from the 1988 classification of probably carcinogenic to humans (Group 2A) 10. A positive association with increased risk of bladder cancer, due to exposure to diesel emissions, was also noted based on limited evidence 10. Gasoline was classified as possibly carcinogenic to humans (Group 2B) based on inadequate evidence in humans and sufficient evidence in experimental animals 10. A meta‐analysis, published before the IARC review, summarizing 35 studies 11, concluded that occupational exposure to diesel emissions was associated with a slightly elevated risk of bladder cancer, with a relative risk in the order of 1.10–1.23 for any exposure and 1.44 for exposure to the highest concentrations of diesel emissions. For most studies, high exposure was defined as 10 or more years of occupational exposure in occupations such as truck or bus driver or heavy equipment operator. An elevated risk of bladder cancer has been reported for many motor exhaust‐related occupations, although findings for most of these studies have been inconsistent 6, 12.

The ubiquitous nature of engine emissions coupled with biological plausibility and the inconsistency of previously published studies emphasize a need for further research. Using data from the National Enhanced Cancer Surveillance System (NECSS), which included detailed lifetime occupational histories and information on a large number of possible cancer risk factors, and an exposure assessment approach that relied on a combination of job‐exposure matrix (JEM) and expert review, we investigated the relationship between both diesel and gasoline engine emissions and bladder cancer in occupationally exposed men.

## Materials and Methods

### Study participants

Case–control data were collected by the NECSS from 1994 to 1997 from participants residing in eight Canadian provinces: British Columbia, Alberta, Saskatchewan, Manitoba, Ontario, Nova Scotia, Newfoundland, and Prince Edward Island. Mailed, self‐administered questionnaires were used to collect detailed information for a number of potential risk factors, for 19 cancer sites, from a national sample of 20,755 incident cancer cases 13. Controls were randomly sampled from the general population of the eight provinces from which cases were selected, using either random digit dialing (Newfoundland and Alberta) or random sampling from provincial health insurance plan databases (British Columbia, Manitoba, Saskatchewan, Nova Scotia, Prince Edward Island). The health insurance plan databases cover more than 95% of the population in the province. Controls were matched to the overall case grouping by sex and 5‐year age group. In total, 2547 male controls returned completed questionnaires, representing 64% of those contacted 14. Of the male bladder cancer cases who received questionnaires, 66% completed them 15.

We restricted our analyses to men ≥40 years of age and who had worked for at least 1 year. This age exclusion was applied to account for the long latency between exposure to environmental carcinogens and induction of cancer 16. Very few bladder cancer cases were under the age of 40 (*N* = 11, 1.6%). For controls to represent the source population from which cases were ascertained, we excluded controls residing in the province of Ontario, as bladder cancer cases were not ascertained for this province. The initial dataset for this analysis included 670 histologically confirmed male bladder cancer cases and 2547 controls. After applying the aforementioned exclusion criteria, 658 incident bladder cancer cases and 1360 controls remained for analyses. Approximately 90% (*n* = 593) of bladder cancer cases were of the urothelial (transitional cell) carcinoma type and 522 of 658 (79%) cases were coded as ICD‐10 code C67.9 (bladder cancer, unspecified). No information was available on cancer stage or grade.

Ethics review board approval for the NECSS study protocol was obtained by all participating provincial cancer registries. All participants provided informed consent. Ethics approval for the exposure assessment work and this analysis was granted by both the University of Toronto and Health Canada Research Ethics Boards.

### Assignment of occupational exposures

The NECSS participants were asked to provide information for each job held, in Canada, for at least 12 months from the time they were 18 years old. This included job title, main tasks, industry, location, and period of employment, as well as information on part‐time, full‐time, and/or seasonal job status. All jobs were assigned a 7‐digit Canadian Classification and Dictionary of Occupation code (1971–1989). Assignment of exposure to diesel engine emissions, gasoline engine emissions, aromatic amines, asbestos, and silica for the control series had recently been carried out by a team of chemists and hygienists based on job descriptions for previous work with the NECSS data 17. To maximize the consistency in exposure assessment across all subjects, a JEM was developed using occupational information from controls as a basis for assigning exposures for bladder cancer cases. The same team of chemists and hygienists who assigned exposure to the control series then reviewed job descriptions for bladder cancer cases, adjusting exposure based on details provided by subjects for each job. The few jobs (11.8%) not covered by the JEM were assessed for exposure manually using the same principles as those applied to the controls. Lastly, for cases and controls, a subset of jobs that were considered to be representative of occupations with exposure to diesel and gasoline emissions were individually reviewed to ensure consistency in exposure assessment. The expert review involved confirming or changing any of the three exposure dimensions assigned by the JEMs, based on specific information in the NECSS database such as job tasks, company or industry name, and era of exposure. The expert review relied on the scientific and technical literature, consultation with external experts, and exposure databases constructed in previous studies.

The expert‐based assessment approach has been previously described in detail by Parent et al. 18. In brief, while the participants' job title was a factor in attributing exposure, the idiosyncrasies of the job activities were taken into account, and there were many examples of participants with the same job title having different exposure profiles; conversely, similar exposures were attributed to many participants with different job titles. Nonexposure was defined as a level that could be encountered in the general environment. Exposure was characterized across three dimensions: reliability, concentration, and frequency according to a semiquantitative three‐point scale of low, medium, and high. Reliability represented the chemist‐hygienist's confidence that exposure was actually present in the job under evaluation. Low reliability referred to a possible exposure, medium to a probable exposure, and high to a definite exposure. Concentration represented the level of intensity of exposure and was assessed on a relative scale according to pre‐established benchmarks and considering ventilation conditions. For instance, for gasoline emissions, low concentration was assigned to jobs such as farmers, medium concentration was assigned to motor vehicle mechanics and repairers working in areas with some ventilation, and high concentration was assigned to motor vehicle mechanics and repairers in poorly ventilated areas. For diesel emissions, low concentration was assigned for jobs such as truck, taxi, and bus drivers in urban areas, medium concentration to jobs such as locomotive operators, and high concentration was assigned to jobs such as garage mechanics maintaining diesel engines in poorly ventilated areas and underground mine workers. Frequency represented the proportion of work‐time exposed and was adjusted for full‐time, part‐time, full‐time seasonal and part‐time seasonal job status. Low corresponded to an exposure frequency of 5% or less of work time, medium between 6% and 30%, and high to more than 30% of the work week. The exposure assessment also took into account the era of employment. In many industries, there has been a shift between gasoline and diesel powered engines over the study period, and these transitions occurred at different times across different industries.

The bladder cancer analysis was based on information collected for 12,367 jobs. For 194 of the 12,367 jobs, there was little or no information in the job title, duties, job type, and location fields and these were coded as missing. These jobs did not contribute information to the overall exposure metrics calculated for the individual. Jobs reported as retirement (*n* = 136), disability (*n* = 6), unemployment (*n* = 5), refugee/prisoner (*n* = 1), volunteer work (*n* = 2), or student (*n* = 64) were coded as unexposed. Exposure assessment was repeated for a random subset of 96 participants with a total of 385 jobs. The kappa statistics for inter‐rater agreement for reliability and concentration was similar, and suggests excellent agreement between exposure assignment (weighted *κ* = 0.81, 0.78–0.85).

### Statistical analysis

Unconditional logistic regression was used to determine the associations between occupational exposure to diesel and gasoline engine emissions and bladder cancer risk in men while controlling for a series of a priori risk factors. Detailed information on family history of cancer, socioeconomic status, and residential history was collected. Cigarette smoking and lifetime environmental tobacco smoke exposure, physical activity, alcohol consumption, coffee, tea, and tap water intake, and dietary information from a food frequency questionnaire were also collected. As information for a large number of variables was available in the NECSS, a screening process was implemented in selecting variables for consideration as possible confounders. A subset of covariates was selected based on a review of the literature and correlation with bladder cancer or exposure to diesel or gasoline engine emissions. A parsimonious model from among this subset was created using stepwise selection with a *P*‐value criterion of 0.15 for both entry and leave in the model. The full model included adjustment for age at interview, proxy respondent, province of residence, cigarette pack‐years, cumulative asbestos, and cumulative silica exposure. Cumulative asbestos and cumulative silica exposures were considered as potential confounders as they occur as co‐exposures with diesel and gasoline engine emissions in occupations such as mining and construction. Cumulative asbestos and cumulative silica exposure were modeled as nominal variables. Cigarette pack‐years were calculated by multiplying average number of cigarettes smoked per day by the number of years smoked, and were used to adjust for duration and intensity of smoking. Environmental tobacco smoke exposure, total and added fat intake, total caloric intake, other aspects of the diet, coffee, tea, alcohol consumption, tap water intake, and the socio‐demographic variables of total education, income, and income adequacy were also considered but were not selected into the final models. Information on participation in various physical activities summarized as total moderate physical activity and total strenuous physical activity in hours per month, and occupational exposure to aromatic amines did not change the effect estimate appreciably (>5%) and were not included in the final models.

Several metrics were constructed to describe occupational exposures to diesel and gasoline engine emissions. These metrics included: ever/never exposed, highest attained concentration of exposure (low, medium, high), highest attained frequency of exposure, duration of exposure in years (categorized into tertiles), duration of exposure at low (tertiles) and high concentrations (cut at median), and a cumulative measure of exposure (tertiles). For all exposure metrics, estimates coded as low reliability were classified as unexposed for the statistical analyses. A total of 125 (20.0%) cases and 224 (16.5%) controls that were exposed but coded as low reliability (possibly exposed) were assumed to have had no exposure.

Concentration was investigated by modeling the highest level of exposure attained by the participants over their lifetime work history. For this categorical variable, each individual was assigned the maximum concentration value identified across all jobs, corresponding to: unexposed, low, medium, and high concentration. Tertiles for duration of exposure at any and low concentrations of diesel and gasoline emissions were based on the distribution in exposed controls. Duration of exposure at high concentrations was also categorized based on the distribution in controls. A three‐level variable was created, with a separate category for the unexposed, and two categories for exposure below and above the median.

Cumulative exposure was defined as:

$$\text{CE}=\sum_{i=1}^{k}C_{i}F_{i}D_{i}$$

where CE is the cumulative exposure, *i* represents the *i*th job held, *k* is the total number of jobs held, C is the concentration of exposure, F is the frequency of exposure adjusted for job status, and D is the duration of employment in years. CE was categorized into four levels as unexposed and tertiles of CE, and is based on the semiquantitative classification described above.

To account for the potentially high correlation between occupational exposure to diesel and gasoline engine emissions, we restricted the analysis to a subset of the population with one exposure but not the other. The confounding effect of each exposure on the other was also evaluated.

Odds ratios (OR) and their 95% confidence intervals were determined by entering each exposure metric into the model one at a time while controlling for confounders. A linear test for trend was conducted by entering each ordinal exposure metric in the model as a continuous variable. All significance tests were two‐tailed, and all analyses were carried out using SAS Version 9.4 (Cary, NC, USA).

## Results

Selected characteristics of male incident bladder cancer cases and controls are presented in Table 1. A total of 658 cases and 1360 controls were included in this analysis. Pack‐years of cigarette smoking exhibited a highly significant, positive dose‐response relationship with bladder cancer in a model adjusted for proxy respondent, province of residence, and age at interview. Being ever exposed to asbestos at work was not associated with increased odds of bladder cancer; however, ever exposure to silica was associated with 1.31 (95% CI: 1.04–1.66) times the odds of bladder cancer compared to unexposed men.

**Table 1:** Select characteristics of male incident bladder cancer cases and controls from the Canadian National Enhanced Cancer Surveillance System, 1994–1997

Characteristic	Cases		Controls		OR a	95% CI	*P*‐value
	*N*	%	*N*	%			
Age at interview							
40–50	52	7.9	137	10.1			
50–60	126	19.2	239	17.6			
60–70	283	43.0	581	42.7			
≥70	197	29.9	403	29.6			
Province of residence							
Newfoundland	42	6.4	105	7.7			
Prince Edward Island	15	2.3	63	4.6			
Nova Scotia	60	9.1	307	22.6			
Manitoba	88	13.4	126	9.3			
Saskatchewan	62	9.4	120	8.8			
Alberta	196	29.8	265	19.5			
British Columbia	195	29.6	374	27.5			
Proxy respondent							
No	405	61.6	902	66.3	1.00		
Yes	253	38.5	458	33.7	1.30	1.06–1.59	0.04
Cigarette pack‐years							
Never smoker	76	11.6	302	22.2	1.00		
0–10	67	10.2	223	16.4	1.15	0.79–1.68	
10–20	120	18.2	233	17.1	1.93	1.37–2.72	
20–30	126	19.2	214	15.7	2.39	1.70–3.38	
30–40	121	18.4	147	10.8	3.54	2.46–5.07	
≥40	137	20.8	217	16.0	2.70	1.91–3.81	
Unknown	11	1.7	24	1.8			
*P*_trend_					<0.001		
Occupational exposure to aromatic amines							
Never	652	99.1	1348	99.1	1.00		
Ever	6	0.9	12	0.9	1.36	0.49–3.79	0.60
Occupational exposure to asbestos b							
Never	619	94.1	1291	94.9	1.00		
Ever	39	5.9	69	5.1	1.18	0.78–1.79	0.43
Occupational exposure to crystalline silica b							
Never	492	74.8	1105	81.3	1.00		
Ever	166	25.2	255	18.8	1.31	1.04–1.66	0.02
Total	658	100.0	1360	100.0			

a Presented odds ratios (OR) are adjusted for age, province of residence, and proxy respondent.

b Binary categories are presented for consistency but models were adjusted for cumulative asbestos and cumulative silica exposure derived from estimates of concentration of exposure, frequency of exposure adjusted for job status, and duration of employment.

There were a total of 12,367 jobs reported by bladder cancer cases and controls in the NECSS, 2772 (22.4%) by cases and 9595 (77.6%) by controls. The average number of jobs held by both cases and controls was ~3 with a minimum of 1 and a maximum of 12 jobs. A total of 430 (15.6%) jobs reported by cases and 1018 (10.8%) jobs reported by controls were coded as probable or certain exposure to diesel engine emissions, and a total of 542 (19.7%) jobs reported by cases and 1673 (17.8%) reported by controls were coded as probable or certain exposure to gasoline engine emissions. In Table 2, jobs considered to be representative of the main occupations entailing exposure to diesel and gasoline emissions are described. Table 2 contains the proportion of men exposed to diesel and/or gasoline engine emissions in these occupations and the most common exposure coding for these occupations. More than 80% of men occupationally exposed to diesel were employed as bus drivers, railway conductors, miners and quarrymen, firefighters, dockworkers, laborers, and foremen. Drivers (taxi, bus, truck, and route), motor vehicle mechanics, commercial travelers, service station attendants, firefighters, dockworkers, fishermen, farmers, and forestry and logging represent occupational groups with a high prevalence of exposure to gasoline engine emissions.

**Table 2:** Proportion of workers exposed to diesel and/or gasoline engine emissions in selected occupations and most common exposure coding, National Enhanced Cancer Surveillance System, 1994–1997

Occupational group	Industry code	% exposed	Gasoline engine emissions			% exposed	Diesel engine emissions		
			Most common exposure coding				Most common exposure coding		
			Confidence	Concentration	Frequency		Confidence	Concentration	Frequency
Motor transport work	9170–9199	85.7	Certain	Low	Medium	68.6	Possible	Low	Medium
Bus drivers	9171	100	Certain	Low	Medium	81.8	Certain	Low	Low
Taxi drivers and chauffeurs	9173	100	Certain	Low	High	0	–	–	–
Truck drivers	9175	100	Certain	Low	Medium	66.4	Certain	Low	Medium
Mechanics	8580–8593	41.5	Certain	Low	High	27.4	Possible	Low	High
Motor vehicle mechanics and repairers	8581	97.3	Certain	Medium	High	21.6	Certain	Medium	High
Printing machinery mechanics	8584	13.7	Possible	Medium	High	61.2	Certain	High	High
Commodity salesmen	5130–5135	27.0	Certain	Low	Low	7.3	–	–	–
Commercial travelers	5133	81.4	Probable	Low	Medium	1.0	–	–	–
Route drivers	5193	100	Certain	Low	High	35.7	Possible	Low	Low
Service station workers	5145	100	Certain	Low	High	72.2	Possible	Low	Low
Railway transport work	9130–9139	0	–	–	–	60.0	Probable	Low	Medium
Locomotive operators	9131	0	–	–	–	68.8	Certain	Medium	High
Railway conductors and brake workers	9133	0	–	–	–	89.5	Certain	Medium	High
Excavators and pavers	8710–8799	3.6	–	–	–	43.0	Possible	Low	Medium
Miners and quarrymen	7710–7719	1.4	–	–	–	92.8	Possible	Low/High	Medium
Firefighters	6111	96.2	Certain	Low	Medium	96.2	Probable	Low	Medium
Dockworkers	9313	100	Possible	Low	Medium	88.9	Certain	Low	Medium
Fishermen	7313	100	Probable	Low	High	79.4	Probable	Low	High
Farming occupations	7111–7199	80.6	Possible	Low	Medium	52.9	Certain	Low	Medium
Material handling equipment operators	9315	66.7	Possible	Low	High	54.2	Possible	Low	High
Forestry and logging occupations	7510–7519	81.9	Certain	High	High	19.0	Probable	Low	Medium
Laborers and foremen	8710–8719	12.1	Probable	Low	Low	96.6	Certain	Medium	High

**Figure 1:**
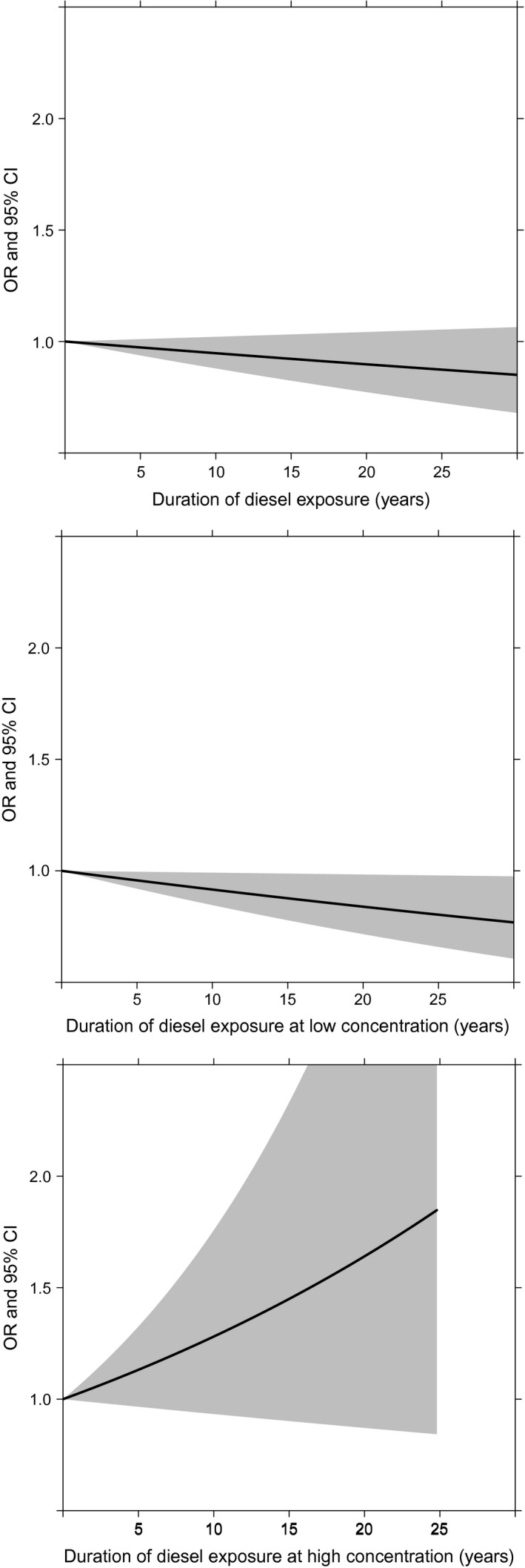
Duration‐response plots for diesel exposure at any (odds ratios [OR] = 1.00, 0.99–1.00), low (OR = 0.99, 0.98–1.00) and high concentration (OR = 1.03, 0.99–1.06). Duration was modeled in years. OR were adjusted for proxy respondent, province of residence, age at interview, cigarette pack‐years, cumulative asbestos, and cumulative silica exposure.

A total of 256 (38.9%) cases and 491 (36.1%) controls were exposed to diesel at some point during their lifetime occupational history. Ever exposure, frequency of exposure, duration of exposure at any concentration and at low concentrations of diesel, and CE were not significantly associated with bladder cancer (Table 3). However, those who had ever been occupationally exposed to medium (OR = 1.46, 1.03–2.08) and high concentrations of diesel engine emissions had elevated odds of bladder cancer (OR = 2.60, 1.47–4.61, *P*_trend_ < 0.01). A significant dose‐response relationship with highest attained concentration of diesel exposure was observed in the minimal model (*P* < 0.01) and exposure to high concentrations of diesel for more than 10 years was associated with 2.94 the odds of bladder cancer compared to unexposed workers (OR = 2.94, 1.36–6.37). However, after adjustment for smoking, and occupational exposure to silica and asbestos these OR were reduced and only the effect estimate for exposure to high concentrations of diesel for >10 years remained statistically significant. In the fully adjusted model, men ever exposed to high concentrations of diesel had 64% greater odds of developing bladder cancer relative to unexposed men (OR = 1.64, 0.87–3.02), but this result was no longer statistically significant. Compared to those who were unexposed, men occupationally exposed to high concentrations of diesel engine emission for >10 years had 2.45 times the odds of bladder cancer (OR = 2.45, 1.04–5.74, *P*_trend_ = 0.07). Exposure‐response curves for duration of exposure at high concentrations show increasing odds of bladder cancer with increasing duration at high concentrations of diesel exposure (OR = 1.03, 0.99–1.06; Fig. 1). Exposure‐response curves for duration of exposure at any (OR: 1.00, 0.99–1.00) and low concentration (OR: 0.99, 0.98–1.00) of diesel exposure do not suggest an association with bladder cancer (Fig. 1).

**Table 3:** Adjusted odds ratios (OR) for bladder cancer in relation to occupational exposure to diesel engine emissions from the Canadian National Enhanced Cancer Surveillance System, 1994–1997

Diesel exposure groups	Cases		Controls		Minimal^a^	*P*‐value	Full^b^	*P*‐value
	*N*	%	*N*	%	OR (95% CI)		OR (95% CI)	
Ever exposed to diesel								
Never	402	61.1	869	63.9	1.00		1.00	
Ever	256	38.9	491	36.1	1.07 (0.88–1.31)	0.51	0.88 (0.70–1.11)	0.29
Highest attained concentration of exposure to diesel								
Unexposed	402	61.1	869	63.9	1.00		1.00	
Low	162	24.6	377	27.7	0.88 (0.70–1.10)	0.26	0.78 (0.60–1.00)	0.05
Medium	66	10.0	89	6.5	1.46 (1.03–2.08)	0.03	1.19 (0.81–1.75)	0.37
High	28	4.3	25	1.8	2.60 (1.47–4.61)	<0.01	1.64 (0.87–3.08)	0.12
*P*_trend_					<0.01		0.49	
Highest attained frequency of exposure to diesel								
Unexposed	402	61.1	869	63.9	1.00		1.00	
<5%	20	3.0	51	3.8	0.88 (0.51–1.52)	0.65	0.77 (0.44–1.36)	0.37
5–30%	133	20.2	270	19.9	0.97 (0.75–1.24)	0.78	0.83 (0.62–1.10)	0.19
≥30%	103	15.7	170	12.5	1.30 (0.98–1.72)	0.07	1.00 (0.74–1.37)	0.98
*P*_trend_					0.22		0.57	
Duration of exposure to diesel								
Unexposed	402	61.7	869	64.4	1.00		1.00	
<7	70	10.7	120	8.9	1.23 (0.89–1.71)	0.21	1.05 (0.74–1.49)	0.79
7–26	80	12.3	164	12.2	1.04 (0.77–1.40)	0.81	0.80 (0.57–1.12)	0.19
≥26	100	15.3	196	14.5	0.99 (0.75–1.31)	0.93	0.83 (0.61–1.15)	0.26
*P*_trend_					0.52		0.29	
Duration of exposure at low concentrations of diesel								
Unexposed	453	69.3	945	69.9	1.00		1.00	
<6	54	8.3	98	7.3	1.22 (0.85–1.74)	0.30	0.98 (0.67–1.43)	0.91
6–26	74	11.3	143	10.6	1.01 (0.74–1.38)	0.96	0.84 (0.60–1.17)	0.31
≥26	73	11.2	166	12.3	0.80 (0.59–1.09)	0.16	0.72 (0.51–1.01)	0.06
*P*_trend_					0.78		0.12	
Duration of exposure at high concentrations of diesel								
Unexposed	630	96.0	1335	98.2	1.00		1.00	
≤10	11	1.7	13	1.0	2.05 (0.86–4.85)	0.10	1.24 (0.52–2.94)	0.63
>10	15	2.3	11	0.8	2.94 (1.36–6.37)	<0.01	2.45 (1.04–5.74)	0.04
*P*_trend_					<0.01		0.07	
Cumulative exposure to diesel								
Unexposed	402	61.7	869	64.4	1.00		1.00	
Lowest tertile	69	10.6	127	9.4	1.14 (0.82–1.57)	0.44	0.96 (0.68–1.36)	0.81
Middle tertile	70	10.7	155	11.5	0.91 (0.66–1.24)	0.55	0.76 (0.54–1.07)	0.11
Highest tertile	111	17.0	198	14.7	1.15 (0.88–1.51)	0.31	0.93 (0.68–1.28)	0.66
*P*_trend_					0.50		0.34	

a Adjusted for proxy respondent, province of residence, and age at interview.

b Adjusted for proxy respondent, province of residence, age at interview, cigarette pack‐years, cumulative asbestos, and cumulative silica exposure.

A total of 315 (47.9%) cases and 577 (42.4%) controls were occupationally exposed to gasoline emissions at some point during their lifetime occupational history. The results of the analysis with occupational exposure to gasoline emissions are presented in Table 4. Ever exposure, highest attained concentration of exposure and duration of exposure at high concentrations of gasoline emissions were not related to bladder cancer in this dataset. The observed OR for highest attained frequency of exposure (OR = 1.44, 1.08–1.91), duration of exposure from six to less than 22 years at any concentration of gasoline (OR = 1.36, 1.03–1.79), five to less than 22 years of exposure at low concentrations of gasoline emissions (OR = 1.34, 1.02–1.78), and the middle tertile of CE to gasoline emissions (OR = 1.31,1.00–1.72) suggest an association with bladder cancer in the minimal model adjusted for proxy respondent, province of residence, and age at interview but these associations disappeared after additional adjustment for cigarette pack‐years and cumulative occupational exposure to asbestos and silica (full model).

**Table 4:** Adjusted odds ratios (OR) for bladder cancer in relation to occupational exposure to gasoline engine emissions from the Canadian National Enhanced Cancer Surveillance System, 1994–1997

Gasoline exposure groups	Cases		Controls		Minimal^a^	*P*‐value	Full^b^	*P*‐value
	*N*	%	*N*	%	OR (95% CI)		OR (95% CI)	
Ever exposed to gasoline								
Never	343	52.1	783	57.6	1.00		1.00	
Ever	315	47.9	577	42.4	1.17 (0.96–1.41)	0.12	1.05 (0.85–1.29)	0.65
Highest attained concentration of exposure to gasoline								
Unexposed	343	52.1	783	57.6	1.00		1.00	
Low	251	38.2	462	34.0	1.14 (0.93–1.40)	0.21	1.05 (0.84–1.30)	0.67
Medium	46	7.0	71	5.2	1.40 (0.94–2.11)	0.10	1.07 (0.68–1.69)	0.76
High	18	2.7	44	3.2	1.04 (0.58–1.85)	0.91	1.01 (0.56–1.85)	0.97
*P*_trend_					0.15		0.71	
Highest frequency of exposure to gasoline								
Unexposed	343	52.1	783	57.6	1.00		1.00	
<5%	12	1.8	42	3.1	0.66 (0.34–1.28)	0.21	0.66 (0.34–1.30)	0.23
5–30%	196	29.3	364	26.8	1.10 (0.88–1.38)	0.40	1.02 (0.80–1.29)	0.89
≥30%	107	16.3	171	12.6	1.44 (1.08–1.91)	0.01	1.21 (0.90–1.63)	0.21
*P*_trend_					0.02		0.33	
Duration of exposure to gasoline								
Unexposed	343	52.8	783	58.1	1.00		1.00	
<6	78	12.0	159	11.8	1.08 (0.79–1.47)	0.64	1.02 (0.74–1.40)	0.92
6–22	112	17.2	177	13.1	1.36 (1.03–1.79)	0.03	1.17 (0.88–1.57)	0.28
≥22	117	18.0	229	17.0	1.06 (0.81–1.38)	0.69	0.97 (0.73–1.28)	0.84
*P*_trend_					0.14		0.67	
Duration of exposure at low concentrations of gasoline								
Unexposed	376	57.9	850	62.8	1.00		1.00	
<5	60	9.2	121	8.9	1.11 (0.79–1.56)	0.55	1.07 (0.75–1.53)	0.70
5–22	114	17.5	176	13.0	1.34 (1.02–1.78)	0.04	1.20 (0.90–1.60)	0.21
≥22	100	15.4	206	15.2	0.97 (0.73–1.28)	0.82	0.91 (0.68–1.23)	0.54
*P*_trend_					0.21		0.61	
Duration of exposure at high concentrations of gasoline								
Unexposed	640	97.3	1316	97.1	1.00		1.00	
≤5	11	1.7	21	1.6	1.18 (0.55–2.53)	0.66	1.21 (0.55–2.66)	0.64
>5	7	1.1	18	1.3	0.99 (0.40–2.44)	0.98	0.89 (0.36–2.23)	0.80
*P*_trend_					0.75		0.85	
Cumulative exposure to gasoline								
Unexposed	343	52.8	783	58.1	1.00		1.00	
Lowest tertile	80	12.3	161	11.9	1.05 (0.78–1.43)	0.74	0.99 (0.72–1.37)	0.96
Middle tertile	116	17.9	186	13.8	1.31 (1.00–1.72)	0.05	1.16 (0.87–1.54)	0.32
Highest tertile	111	17.1	218	16.2	1.10 (0.84–1.44)	0.49	0.99 (0.74–1.32)	0.92
*P*_trend_					0.17		0.75	

a Adjusted for proxy respondent, province of residence, and age at interview.

b Adjusted for proxy respondent, province of residence, age at interview, cigarette pack‐years, cumulative asbestos, and cumulative silica exposure.

The results of a sensitivity analysis restricting for one exposure, either gasoline or diesel but not both, to a subset with no exposure to the other are presented in Table 5. There were 70 (20.4%) case and 139 (17.8%) control men who had been exposed to diesel exhaust at some point during their working history but had never been exposed to gasoline engine emissions. While not statistically significant, the OR for exposure to high concentrations of diesel exhaust and duration of exposure at high concentrations of diesel for more than 10 years still suggested an increased risk of bladder cancer. Restricting to a subset of the population with no exposure to diesel emissions revealed significantly greater odds of bladder cancer for men who were exposed to gasoline engine emissions for more than 30% of work time (high frequency) at some point during their occupational history (OR = 1.59, 1.04–2.43) compared to unexposed men in the fully adjusted model. The highest tertile of CE to gasoline was associated with 1.66 times the odds of bladder cancer compared to unexposed (OR = 1.66, 0.98–2.80).

**Table 5:** Adjusted odds ratios (OR) for bladder cancer in relation to diesel and gasoline engine emissions from a restricted analysis for one exposure to a subset with no exposure to the other substance, Canadian National Enhanced Cancer Surveillance System, 1994–1997

Exposure groups	Cases		Controls		Diesel	*P*‐value	Cases		Controls		Gasoline	*P*‐value
	*N*	%	*N*	%	OR (95% CI)^a^		*N*	%	*N*	%	OR (95% CI)^a^	
Ever exposed												
Never	273	79.6	644	82.3	1.00		273	67.9	644	74.1	1.00	
Ever	70	20.4	139	17.8	0.94 (0.65–1.38)	0.77	129	32.1	225	25.9	1.17 (0.88–1.54)	0.27
Highest attained concentration												
Unexposed	273	79.6	644	82.3	1.00		273	67.9	644	74.1	1.00	
Low	27	7.9	86	11.0	0.68 (0.41–1.12)	0.13	102	25.4	176	20.3	1.17 (0.86–1.58)	0.32
Medium	29	8.5	37	4.7	1.36 (0.76–2.42)	0.30	18	4.5	27	3.1	1.26 (0.63–2.54)	0.51
High	14	4.1	16	2.0	1.45 (0.61–3.44)	0.40	9	2.2	22	2.5	1.05 (0.46–2.41)	0.91
*P*_trend_					0.38						0.38	
Highest frequency of exposure												
Unexposed	273	79.6	644	82.3	1.00		273	67.9	644	74.1	1.00	
<5%	4	1.2	9	1.2	0.84 (0.23–3.06)	0.79	6	1.5	17	2.0	0.86 (0.32–2.30)	0.77
5–30%	25	7.3	57	7.3	0.87 (0.50–1.50)	0.61	72	17.9	143	16.5	1.02 (0.72–1.43)	0.93
>30%	41	12.0	73	9.3	1.02 (0.63–1.64)	0.94	51	12.7	65	7.5	1.59 (1.04–2.43)	0.03
*P*_trend_					0.12						0.12	
Duration of exposure												
Unexposed	273	80.1	644	83.0	1.00		273	68.3	644	74.7	1.00	
Lower tertile	23	6.7	39	5.0	1.17 (0.64–2.11)	0.61	46	11.5	84	9.7	1.10 (0.73–1.67)	0.64
Middle tertile	20	5.9	53	6.8	0.65 (0.35–1.19)	0.16	47	11.8	82	9.5	1.13 (0.75–1.70)	0.57
Higher tertile	25	7.3	40	5.2	1.16 (0.65–2.07)	0.63	34	8.5	52	6.0	1.36 (0.84–2.20)	0.21
*P*_trend_					0.26						0.26	
Duration of exposure at low concentrations												
Unexposed	307	89.5	687	88.3	1.00		292	73.0	675	77.9	1.00	
Lower tertile	11	3.2	29	3.7	0.66 (0.31–1.42)	0.29	37	9.3	60	6.9	1.30 (0.82–2.05)	0.27
Middle tertile	12	3.5	37	4.8	0.60 (0.29–1.24)	0.17	46	11.5	91	10.5	0.95 (0.63–1.43)	0.81
Higher tertile	13	3.8	25	3.2	0.99 (0.47–2.09)	0.98	25	6.3	40	4.6	1.27 (0.74–2.19)	0.39
*P*_trend_					0.44						0.44	
Duration of exposure at high concentrations												
Unexposed	329	96.2	767	98.1	1.00		393	97.8	847	97.9	1.00	
Below median	4	1.2	6	0.8	0.86 (0.22–3.41)	0.82	4	1.0	9	1.0	1.09 (0.31–3.83)	0.89
Above median	9	2.6	9	1.2	1.96 (0.69–5.54)	0.20	5	1.2	9	1.0	1.15 (0.37–3.60)	0.80
*P*_trend_					0.79						0.79	
Cumulative exposure												
Unexposed	273	80.1	644	83.0	1.00		273	68.3	644	74.7	1.00	
Lower tertile	15	4.4	35	4.5	0.87 (0.45–1.71)	0.69	51	12.8	93	10.8	1.08 (0.73–1.60)	0.71
Middle tertile	21	6.2	42	5.4	1.02 (0.55–1.87)	0.96	45	11.3	87	10.1	1.06 (0.70–1.60)	0.78
Higher tertile	32	9.4	55	7.1	0.97 (0.57–1.65)	0.91	31	7.8	38	4.4	1.66 (0.98–2.80)	0.06
*P*_trend_					0.12						0.12	

a Adjusted for proxy respondent, province of residence, age at interview, cigarette pack‐years, cumulative asbestos, and cumulative silica exposure.

## Discussion

While diesel engine emissions are a cause of lung cancer, based on the consensus decision by IARC, evidence for other cancer sites is limited and inconsistent 10. Frequency of exposure and duration of exposure at low concentrations of diesel emissions were not associated with bladder cancer. However, while the positive association was attenuated and the result was no longer statistically significant after adjusting for a recognized risk factor (smoking) and occupational co‐exposures, the magnitude of the association suggests an increased risk of bladder cancer with exposure to high concentrations of diesel emissions. We also observed a significantly elevated risk of bladder cancer with duration of employment for >10 years in occupations with exposure to high concentrations of diesel engine emissions, even after taking into account smoking and other occupational risk factors.

Many studies have used job or industry title as a proxy for diesel emissions exposure. Results of a meta‐analysis of these types of studies 11 suggest an increased risk of bladder cancer for heavy equipment operators, truck drivers, and bus drivers. More recent studies have also reported associations with diesel emissions‐related occupations including drivers, mechanics, mining, and heavy equipment operators 6, 15, 19, 20, 21, 22. A meta‐analysis of JEM‐based studies 11 corroborates our observation of a positive association between diesel engine emissions and bladder cancer risk. However, four studies published since this meta‐analysis that used comprehensive exposure assessment methods did not observe an association with bladder cancer 20, 23, 24, 25. Two of these studies based exposure assessment on industry of employment as reported in the population census and did not have detailed information on job tasks and location 20, 23. One was likely underpowered to detect an association as they had exposure information for 200 cases and 385 controls 25. The other reported an elevated but nonsignificant risk for diesel exhaust exposure in the study population overall and a significantly increased risk of bladder cancer among current or former smokers who smoked more than 15 cigarettes a day 24.

After adjustment for confounding factors, we did not see associations with any of the gasoline engine emissions exposure metrics, suggesting that after accounting for smoking and occupational co‐exposures, men exposed to gasoline engine emissions in the workplace are unlikely to have a significantly elevated risk of bladder cancer compared to unexposed workers. However, in an analysis restricted to men who were never occupationally exposed to diesel engine emissions, we observed results that are more consistent with an exposure‐response; an association with high frequency of exposure and the highest tertile of CE to gasoline. This restriction was applied to take into account the correlated nature of exposures to diesel and gasoline emissions (*r* = 0.429, *P* < 0.0001). However, it is possible that the observed associations are due to chance as the sensitivity analysis included a smaller number of participants, and the existing literature is not supportive of an association between gasoline engine emissions and an increased risk of cancer. In previous lung cancer studies 17, 18, 26, investigators did not observe an association with exposure to gasoline engine emissions. Very few studies have been published on the relationship with bladder cancer; however, Guo et al. 20, reported a slightly elevated risk of bladder cancer at low exposure to gasoline engine emissions, mainly attributable to drivers. They also reported an association with lung cancer in women, but not in men. However, their results suggest that the excess risk observed for gasoline emissions may be due to inadequate control for confounding by smoking. Some have pointed out that it may be difficult to observe an association for gasoline engine emissions because of a lack of an appropriate referent group due to the ubiquitous nature of gasoline emissions in nonoccupational settings 18.

Our results for exposure to diesel engine emissions are consistent with the hypothesis of a threshold effect—an excess risk of bladder cancer at high concentrations of exposure only. The mechanism underlying this relationship remains speculative; particles deposited in the lungs, and their metabolites, can usually be found in measurable quantities in other organs 27. It is postulated that the gases and particulate matter, which include elemental carbon and PAHs, emitted by diesel engines 16, and benzene and ethylene dibromide emitted by gasoline engines 17 are inhaled and deposited in the lungs 18. Subsequent clearance by mucociliary transport and diffusion into the pulmonary capillaries is likely the pathway by which particles enter the bloodstream and translocate to other organs 27. This may lead to an accumulation of related metabolites in the urine, where they may interact with the urothelium of the bladder to initiate carcinogenesis 28. Higher levels of diesel metabolites have been observed in the urine of exposed individuals compared to unexposed individuals 29, 30, 31, 32, 33, 34, 35. These metabolites can cause genotoxic effects such as DNA damage and DNA‐adduct formation 36, 37 in urothelial cells, which can lead to cancer if the damage is not repaired. Studies in experimental models indicate that a large single dose of exposure to diesel particles has a more pronounced and sustained effect on DNA damage than the effect of the same total dose administered over the course of several days 36. This suggests that there may be a threshold for the genotoxic effect of diesel exhaust particles. Additionally, excreted urinary carcinogens may also promote carcinogenesis indirectly by damaging the epithelium and promoting cell proliferation 38. However, it is also possible that like with gasoline engine emissions, the relationship at lower exposure levels is harder to detect because diesel emissions are also ubiquitous in the environment. The difference in level of exposure between the unexposed, defined as the exposure present in the general environment, and those occupationally exposed at low concentrations may confer a smaller increase in cancer risk that is harder to detect by epidemiological studies utilizing retrospective exposure assessment methods 39.

Engine emissions are complex mixtures and vary in composition depending on engine type, age and operating conditions, the fuel and lubricating oil used, and presence or absence of an emissions control system 40. The complexity of engine emissions makes it difficult to isolate a specific component that may contribute to increased cancer rates. Organic compounds from diesel and gasoline engines are qualitatively similar but there are quantitative differences. Older, light‐duty diesel engines emit 50–80 times more particulate matter and heavy‐duty diesel engines emit 100–200 times more particulate matter than catalytically equipped gasoline engines 9, 41, 42. With increasingly stringent regulations and advances in emission control technologies, this difference has decreased in newer diesel engines. Gasoline engines without catalytic converters produce a similar quantity of PAHs as diesel engines 26. Furthermore, changes in technology, workplace practices, and regulation mean that occupational exposure to diesel and gasoline engine emissions varies over time 43. Thus, for population‐based studies that retrospectively assess exposure it is impractical and often impossible to obtain quantitative exposure measurements. Semiquantitative estimates, accounting for era of exposure, are often assumed to be more credible for these types of studies 44. However, there are limitations of this approach in assessing exposure.

A limitation of semiquantitative estimates of exposure is that it assumes that all subjects within a group have the same exposure and that relationships between exposure groups are represented by the values assigned to the exposure categories. In reality, variability in exposure at worksites is greater than these differences assume. Relative, semiquantitative estimates of exposure can potentially introduce nondifferential misclassification of exposure, which can reduce power and attenuate observed effect estimates. Exposure estimates of lower confidence in particular can be a source of error. For this reason, we assigned a reliability score to all exposure values and grouped exposure estimates scored as low reliability with the unexposed. A sensitivity analysis demonstrated that including those with lower confidence did not change the conclusions of this study. In most cases including “possibly exposed” workers widened the confidence interval around a point estimate because of higher error.

A further limitation of this study is the use of self‐reported data for lifetime occupational histories. Inaccuracies in recall of job duration, tasks, or any other component used to assign exposure may have contributed to misclassification as the ability of respondents to recall details of job tasks performed in the distant past may be limited. Nevertheless, we expect that the resulting misclassification would be nondifferential in nature, and would most likely attenuate the observed associations. Case–control studies are susceptible to recall bias as cases may be more likely to recall past exposures and details of their employment history than controls. This is particularly of concern when data are self‐reported; however, this bias is largely reduced when exposure is based on assignment of exposures from a lifetime occupational history by expert assessment or application of a JEM 44.

Finally, the response rates in this study were modest for the time period during which recruitment occurred. Although selection bias resulting from lower response rates is a concern, evidence suggests that the magnitude of this bias is small in most epidemiologic studies and participation rates alone do not determine the extent of this bias 45. It is unlikely that participation in the study is directly related to diesel and gasoline emissions exposure, since this was not identified as a study objective during recruitment. The potential for selection bias in our analysis was reduced because very few jobs (194, 1.6%) were excluded from the exposure assessment, and this proportion did not differ between cases and controls. Additionally, the observed relationships with smoking and age are in the direction and of a magnitude that is expected based on results from other studies, and socioeconomic status did not have a large impact on risk estimates.

Participants flagged as proxy respondents received assistance in completing their questionnaires, which includes, but is not limited to true proxy respondents. In some cases, the participants themselves provided the information but were assisted with recording their responses. Although not shown in this article, analyses restricted to self‐respondents, excluding proxy respondents, yielded results similar to those from the main analyses.

Despite the limitations outlined above, our study has several important strengths. The population‐based nature of this study allowed us to evaluate risks across a wide range of exposure levels and circumstances, which is not the case in industry‐specific studies with typically high and homogeneous exposure patterns. Additionally, because the study was based on different occupational groups, the likelihood for confounding by occupational exposures that would occur at high frequency within a given industry is reduced.

A further strength was the availability of lifetime occupational histories which were used by a team of chemists and hygienists to assign exposures on a case‐by‐case basis, based on the individual job descriptions, taking into consideration factors such as the era of exposure, work practices, and work location among others. While it is difficult to validate retrospective exposure assessment, we have shown that self‐reported job histories are valid 46, that the exposure assessment approach employed is reliable 47, 48 and, in a limited trial, that the exposure assessment approach reflects past measured exposures 49. Furthermore, this method is widely considered as the reference method of exposure assessment for retrospective studies 50.

The NECSS also collected information on a comprehensive listing of suspected bladder cancer risk factors allowing us to take into account their potential confounding influence. This included occupational exposure to aromatic amines, asbestos, and silica, a good measure of both personal and environmental tobacco smoke exposure and other behavioral risk factors for bladder cancer. Additionally, a large number of bladder cancer cases and controls were available in the NECSS, which meant we had excellent power to detect associations. Occupational studies that use job title as a proxy for exposure often use “office workers” or another presumably unexposed occupational group as the referent group; however, we had an internal unexposed comparison group, providing a more valid estimate of association.

In summary, we investigated the role of concentration, duration, and CE to engine emissions on bladder cancer risk, and had access to full occupational and smoking histories. The findings of this study extend the epidemiologic literature on the role of diesel emissions in occupational carcinogenesis and support the hypothesis that occupational exposure to diesel engine emissions is an occupational risk factor for bladder cancer. Our results also suggest that the frequency of exposure to gasoline engine emissions may be related to bladder cancer.

## Conflict of Interest

None declared.
